# UV Degradation and Recovery of Perovskite Solar Cells

**DOI:** 10.1038/srep38150

**Published:** 2016-12-02

**Authors:** Sang-Won Lee, Seongtak Kim, Soohyun Bae, Kyungjin Cho, Taewon Chung, Laura E. Mundt, Seunghun Lee, Sungeun Park, Hyomin Park, Martin C. Schubert, Stefan W. Glunz, Yohan Ko, Yongseok Jun, Yoonmook Kang, Hae-Seok Lee, Donghwan Kim

**Affiliations:** 1 Korea University, Department of Materials Science and Engineering, Seoul, 136-713, Republic of Korea; 2 Fraunhofer Institute for Solar Energy Systems ISE, Freiburg, 79110, Germany; 3 University Freiburg, Laboratory for Photovoltaic Energy Conversion, Freiburg, 79110, Germany; 4 Konkuk University, Department of Materials Chemistry and Engineering, Seoul, 143-701, Republic of Korea; 5 KU·KIST Green School, Graduate School of Energy and Environment, Korea University, Seoul, 136-713, Republic of Korea

## Abstract

Although the power conversion efficiency of perovskite solar cells has increased from 3.81% to 22.1% in just 7 years, they still suffer from stability issues, as they degrade upon exposure to moisture, UV light, heat, and bias voltage. We herein examined the degradation of perovskite solar cells in the presence of UV light alone. The cells were exposed to 365 nm UV light for over 1,000 h under inert gas at <0.5 ppm humidity without encapsulation. 1-sun illumination after UV degradation resulted in recovery of the fill factor and power conversion efficiency. Furthermore, during exposure to consecutive UV light, the diminished short circuit current density (J_sc_) and EQE continuously restored. 1-sun light soaking induced recovery is considered to be caused by resolving of stacked charges and defect state neutralization. The J_sc_ and EQE bounce-back phenomenon is attributed to the beneficial effects of PbI_2_ which is generated by the decomposition of perovskite material.

Power conversion efficiency (PCE) of orreganic-inorganic hybrid perovskite solar cells has increased from 3.81% to 22.1% in just 7 years^1^,^2^. In 2009, Kojima *et al*. reported the first application of CH_3_NH_3_PbI_3_ and CH_3_NH_3_PbBr_3_ perovskites as sensitizers for photovoltaic devices^1^. Perovskite absorbers have an ABX_3_ crystal structure^3^,^4^,^5^, usually composed of an organic material (A site), a metal (B site), and a halide (X site). The A site is usually occupied by methylammonium (CH_3_NH_3_), formamidinium (HC(NH_2_)_2_), or a combination of both materials. Recently, the addition of cesium (Cs) and guanidinium (Gu) has been reported^6^,^7^,^8^,^9^. Generally, the B site is occupied by metals (e.g., lead (Pb) or tin (Sn)), while the X site is occupied by halides such as iodine (I), bromine (Br), or chlorine (Cl). This organic-inorganic hybrid material has a number of beneficial characteristics that render it suitable for photovoltaic applications. For example, a high absorption coefficient (~10^5^ cm^−1^)^10^,^11^,^12^,^13^,^14^, long diffusion length (~1 μm)^15^,^16^, direct band gap, and multiple fabrication methods^15^,^17^,^18^,^19^. Development of all solid-state perovskite solar cells containing Spiro-MeOTAD, optimization of the fabrication processes, device structures, and material substitution/addition have been investigated to obtain higher PCE^10^,^17^,^18^,^19^,^20^,^21^. In addition, because of the tunable band-gap^22^,^23^ and simple fabrication steps, perovskite solar cells are an attractive candidate for tandem applications, enabling >30% efficiency potential^24^,^25^,^26^,^27^.

Despite such attractive properties, a number of challenges prevent the commercialization of perovskite solar cells, such as the lack of stability, use of Pb, and scale-up issues. Although the replacement of Pb with Sn or other materials is of particular interest, Sn-based perovskite solar cells show even lower stability than Pb-based perovskite congeners^28^,^29^,^30^. Issues regarding scale-up have been addressed by the development of evaporation^19^, doctor blade^31^, roll-to-roll^32^, and inkjet printing^33^ processes; however, stability problems still need to be solved. According to previous literature reports, the stability of perovskite solar cells is influenced by four main factors: moisture^34^,^35^,^36^, heat^37^,^38^, voltage^39^, and UV light^34^,^40^, with moisture being the most critical factor. Attempts to improve the stability of perovskite solar cells have focused on encapsulation^40^,^41^, replacement/substitution of selective contacts^42^,^43^, interlayer insertion^44^,^45^, development of novel cell and module configurations^46^,^47^, and modification of the perovskite light-absorbing material^6^,^23^,^42^,^48^,^49^,^50^. Nevertheless, issues relating to stability have still not been resolved, and therefore, further studies must be carried out.

In particular, stability upon UV light exposure (hereafter, UV stability), the photocatalytic effect of TiO_2_ is discussed as a main reason of perovskite degradation. Niu *et al*. reported that perovskite underwent degradation upon UV irradiation in the presence of both moisture and oxygen^34^. Snaith *et al*. then reported enhanced UV stability with UV filter or upon substitution of TiO_2_ with Al_2_O_3_^40^. In addition, Ito *et al*. identified the interface between the perovskite and the mesoporous TiO_2_ scaffold as the area of cell degradation commencing, reporting enhanced stability with the incorporation of an Sb_2_S_3_ interlayer at the TiO_2_/perovskite interface^45^. All of these studies have been conducted to under moisture- and oxygen-containing atmosphere with AM1.5G (1-sun) full solar spectrum irradiation. However, under such conditions, determination of the effects of UV light alone on perovskite degradation is challenging, since all wavelengths of light are employed and perovskite solar cells are particularly sensitive to moisture.

Herein, to investigate the effects of UV light alone on the degradation of perovskite solar cells, UV stability experiments were conducted in a glove box (<0.5 ppm average humidity, Ar atmosphere, 25 °C), wherein perovskite solar cells were exposed to 365 nm UV light over the course of 1,000 h under open circuit condition. The power of the UV light employed in this study was approximately 7.6 mW·cm^−2^, giving a UV intensity approximately 1.5 times higher than that in the AM1.5G 100 mW·cm^−2^ solar spectrum, which has a UV intensity of only 4.6 mW·cm^−2^ at wavelengths below 400 nm^44^. Continuous degradation of perovskite solar cell performance was observed even in the absence of moisture, oxygen, and longer wavelength light. Interestingly, UV-degraded FF and PCE of the perovskite solar cell were recovered upon subsequent 1-sun illumination. In case of the J_sc_ and EQE, rapidly decreased values bounced back continuously with consecutive UV light exposure. The processes involved in UV degradation and recovery of the perovskite solar cells were characterized by UV-visible spectroscopy, X-ray diffraction (XRD), light current-voltage (LI-V), external quantum efficiency (EQE), Electrochemical Impedance Spectroscopy (EIS), μ-photoluminescence spectroscopy (μ-PLS), and μ-light beam induced current (μ-LBIC) analyses.

## Results

### UV degradation under inert gas and beneficial effect of degradation by-product PbI_2_

**Figure 1:**
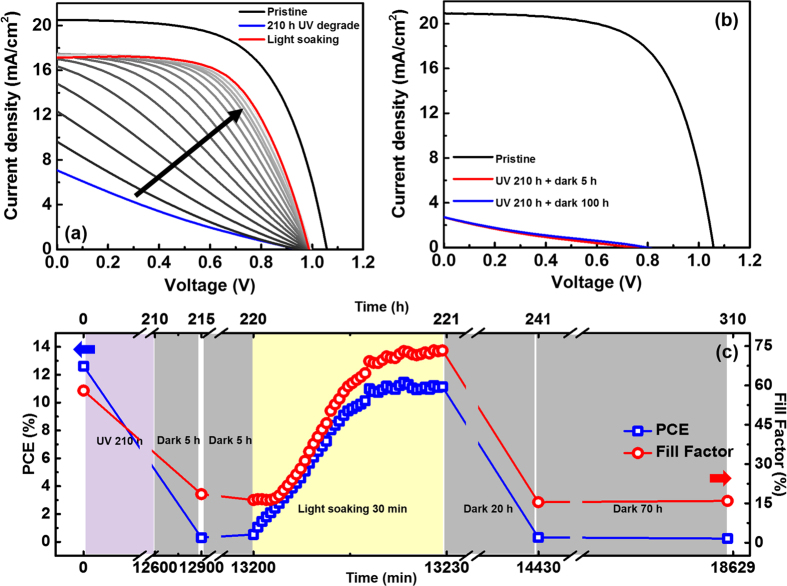
Light-IV curve of (**a**) a pristine device, 210 h UV exposed, during 15 min light soaking and (**b**) pristine device, 210 h UV exposed and then rested in dark under inert gas atmosphere for 5 h and 100 h. (**c**) PCE and FF of a device with denoted light conditions.

**Figure 2:**
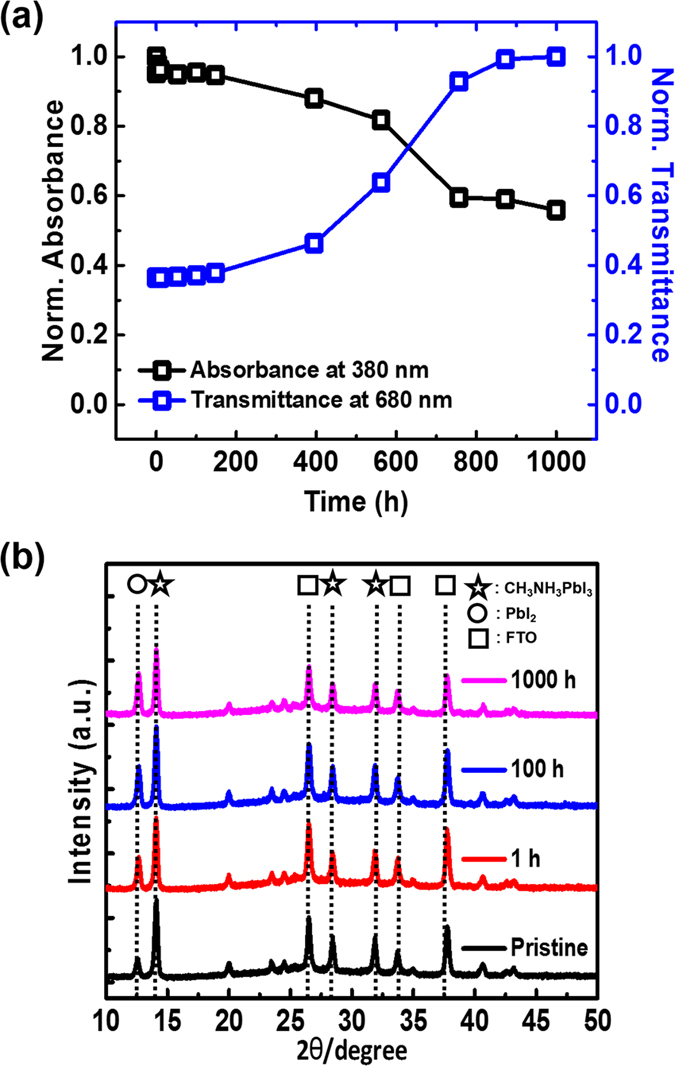
(**a**) Normalized light absorbance (at 380 nm) and transmittance (at 680 nm). (**b**) X-ray diffraction patterns of the devices following UV exposure.

Figure 1a shows LI-V curves of 210 h UV exposed perovskite solar cell under inert gas atmosphere at open circuit and 1-sun light soaking result. Although UV light was exposed under inert gas atmosphere, UV exposure made significant degradation on perovskite solar cells. However, UV degradation was recovered by 1-sun light soaking. Initially 12.2% efficiency was degraded to 1.36% during UV exposure and recovered to 10.4% with continuous 1-sun light soaking. During recovery process, there was no significant change in XRD peaks (Supplementary Fig. S1). On the other hand, UV degradation was not recovered when device rested in dark as shown in Fig. 1b. 1-sun light soaking induced recovery was also occurred after 10 h delay in dark. Figure 1c shows PCE and FF values when devices exposed to UV light, 1-sun light, and rested in dark. When device was rested in dark after 1-sun light soaking recovery, device performance was retrogressed to degraded state. The J_sc_ and open circuit voltage (V_oc_) show similar tendency (Supplementary Fig. S2a,b). To investigate detail steps of UV degradation and recovery, new set of devices was exposed to UV light during 1,000 h and characterized at specific times. Figure 2 shows the normalized light absorbance and X-ray diffraction patterns of UV exposed perovskite devices for different times.

During UV exposure under Ar atmosphere, the light absorption of the perovskite device started to decrease after 200 h, accompanied by increase in transmittance. This can be understood as perovskite materials started to lose their ability to absorb light after 200 h UV exposure. Full absorbance and transmittance data are presented in Supplementary Fig. S3. The X-ray diffraction patterns presented in Fig. 2b show several strong peaks, where the peaks at 14.1°, 28.5°, and 31.9° can be assigned to the (110), (220), and (310) planes of the CH_3_NH_3_PbI_3_ perovskite. 12.6° peak corresponded to traces of PbI_2_ that remained during device fabrication, which is in agreement with the results of previous studies^51^. The remaining peaks corresponded to the FTO substrate^21^,^44^,^51^. With increasing UV exposure time, the ratio between the PbI_2_ (12.6°) and CH_3_NH_3_PbI_3_ (14.1°) peaks increased and this means CH_3_NH_3_PbI_3_ perovskite decomposing to PbI_2_ continuously^34^,^44^,^45^.

**Figure 3:**
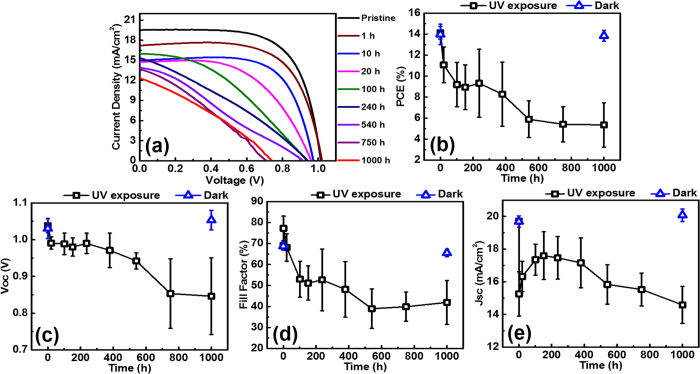
(**a**) Light I-V curve following the denoted UV exposure times. Variation in (**b**) PCE, (**c**) V_oc_, (**d**) FF, and (**e**) J_sc_ with increasing UV exposure time (error bars are shown). All values are average obtained for six cells. The blue triangular symbol denotes the measurement for another device stored in the dark for 1,000 h. Perovskite solar cells were tested at the indicated UV exposure times. IV-measurement was conducted under AM1.5G 100 mW·cm^−2^ illumination. The voltage time setting was 200 ms, the active area was 0.125 cm^2^, and a 0.075 cm^2^ mask was used. Measurements were carried out in the open circuit voltage to short circuit voltage direction (i.e., reverse direction). For the measurement, devices were removed from the glove box and characterization was conducted under air at room temperature. A relative humidity of approximately 25% was maintained. During measurement, there was continuous increase in solar cell parameters as discussed later. Here, saturated values are presented.

Figure 3 and Table 1 show multiple light I-V measurements acquired during 1,000 h UV exposure under open circuit conditions. The parameters, summarized in Fig. 3 and Table 1, are the fully recovered values with 1-sun light soaking as indicated in Fig. 1. In Fig. 3a,b, UV degradation began immediately after 1 h of UV exposure and significant degradation occurred at 100 h. From 100–200 h, PCE degradation retarded because of the increasing J_sc_. 200–1,000 h, there was a continuous degradation in device performance with a decrease in V_oc_, J_sc_, and FF. Exposure to UV for 1,000 h led to 65% degradation of the PCE, while the FF exhibited the most pronounced degradation during this period. As shown in Fig. 3c, V_oc_ underwent little degradation before 400 h, except for the initial degradation. But V_oc_ subsequently dropped to 0.8 V during 400–1,000 h. Figure 3e shows an unexpected behaviour of the J_sc_. Initial 20 h of the exposure made sharp fell of J_sc_. However, after rapid degradation, current bounce back reaching almost 90% of the initial value was observed. This phenomenon can be accounted for beneficial effects of the degradation by-product PbI_2_. The beneficial effect of PbI_2_ on perovskite solar cells has been described in recent literatures^51^,^52^,^53^,^54^,^55^,^56^ Supasai *et al*. reported the passivation effect of PbI_2_ and reduced defect states following PbI_2_ generation^54^. In addition, Chen *et al*. reported that PbI_2_ can passivate recombination sites at both the perovskite grain boundary and the perovskite/TiO_2_ interface, thus improving the electrical properties of the device^52^. Also Kim *et al*. reported greatly improved solar cell performance and reduced hysteresis in the presence of PbI_2_^53^.

Consequently, UV degradation and recovery process can be explained by generation of traps (defects, charge stack) and simultaneous passivation and neutralization of these traps by PbI_2_, which is generated as degradation by-product of perovskite. Initial 10 h UV exposure will produce abundant traps. These traps bring rapid fell of all solar cell parameters as shown in Fig. 3. However, in 10–200 h, as UV light degradation progress, PbI_2_ would be generated. This PbI_2_ will continuously passivate existing traps and enhance electron extraction. As a result of traps passivation, the V_oc_ slightly increased with retarded degradation. Also, enhanced electron extraction will produce the J_sc_ bounce back. Meanwhile, the FF continuously decreased because PbI_2_ is not conductive materials. As UV degradation progressed further, much more PbI_2_ will be generated and after a certain point, the cell parameters decreased again, as shown in Fig. 3 200–1,000 h. Improved electron extraction with PbI_2_ passivation is verified by EIS measurement and possible energy band structure change is shown in Supplementary Fig. S4. Increased recombination resistance observed with 200 h UV exposure times.

### Current bounce back during UV degradation

**Figure 4:**
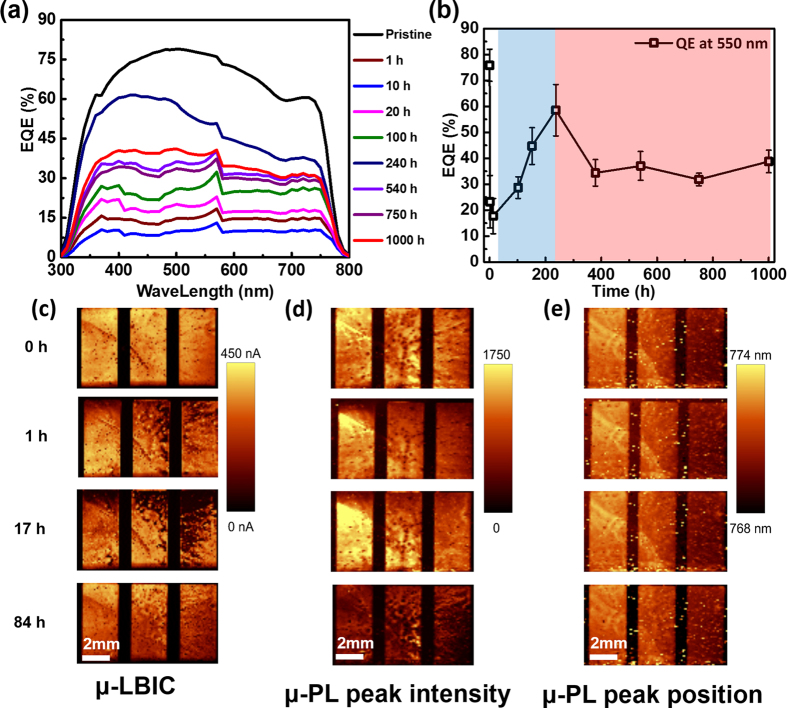
(**a**) EQE at 300–800 nm, and (**b**) at 550 nm for devices subjected to UV exposure for times in the range 0–1,000 h. Results are an average of data for six samples. Spatially resolved results: (**c**) μ-LBIC, (**d**) μ-PL peak intensity in arbitrary units, and (**e**) peak position in nm. Three single cells, which are located on one glass substrate, are depicted (ea. 0.125 cm^2^). The cells were illuminated from the glass side using a 640 nm laser.

The J_sc_ bounce back phenomenon was also found in EQE measurements. Figure 4a shows the variation in the EQE of the perovskite solar cells upon exposure to UV light. The EQE followed a similar tendency to that previously described for a J_sc_ (Fig. 3e), with almost 70% of the initial loss being recovered. This can be more clearly observed in Fig. 4b, which shows the variation in the EQE at 550 nm.

At the first period of UV degradation, the J_sc_ and EQE decreased rapidly. Initial rapid decrease is not directly due to CH_3_NH_3_PbI_3_ decomposition. As shown in Fig. 2a,b, light absorbance and CH_3_NH_3_PbI_3_ (peak at 14.1°) shows almost no change during the first period of UV degradation. This is more likely to be due to the changes of interfaces in the perovskite device like trap formation. Traps at interfaces may block electron extraction by capturing carriers and induce lowered current. In the second period (20–250 h), the J_sc_ and EQE underwent a bounce back. This can be attributed to the beneficial effects of the degradation by-product PbI_2_. As mentioned previously, the generated PbI_2_ can passivate recombination sites and improve the carrier extraction^51^,^52^,^53^,^54^,^55^. In the third period (250–1,000 h), the J_sc_ and EQE decreased once again due to further degradation of CH_3_NH_3_PbI_3_, with a resulting decrease in the perovskite light absorbance and device performance.

To gain insight into the current bounce-back phenomenon, spatially resolved μ-LBIC and μ-PLs measurements were obtained by analysing a new set of devices with Au electrodes. The μ-LBIC (Fig. 4c) data generally corroborate the EQE and I-V measurements, exhibiting both degradation and recovery within the 84 h. The peak intensity (Fig. 4d), which is a function of the carrier density, changed significantly during the investigation. It decreases considerably after 84 h, as indicated by dark (low PL intensity) regions on the entire device. In contrast to the peak intensity, analysis of the spectral position of the PL peak (Fig. 4e) indicates that the bandgap of the absorber material is not affected by UV exposure, as also confirmed by the absorbance and XRD data shown in Fig. 2. Based on the fact that PL intensity is either caused by radiative recombination, at least until 17 h UV exposure, CH_3_NH_3_PbI_3_ perovskite was not degraded much. However, between 17–84 h, PL intensity decreased significantly and this means lower radiative recombination or higher non-radiative recombination (trap-assisted). This also confirm that initial degradation of perovskite device was not mainly because of CH_3_NH_3_PbI_3_ material degradation. Until now it is not clear how the interfaces influence the degradation/recovery mechanism as the used characterization methods yield no information about that at the moment, but this will be addressed in following work.

### UV degradation and 1-sun light soaking induced recovery

**Figure 5:**
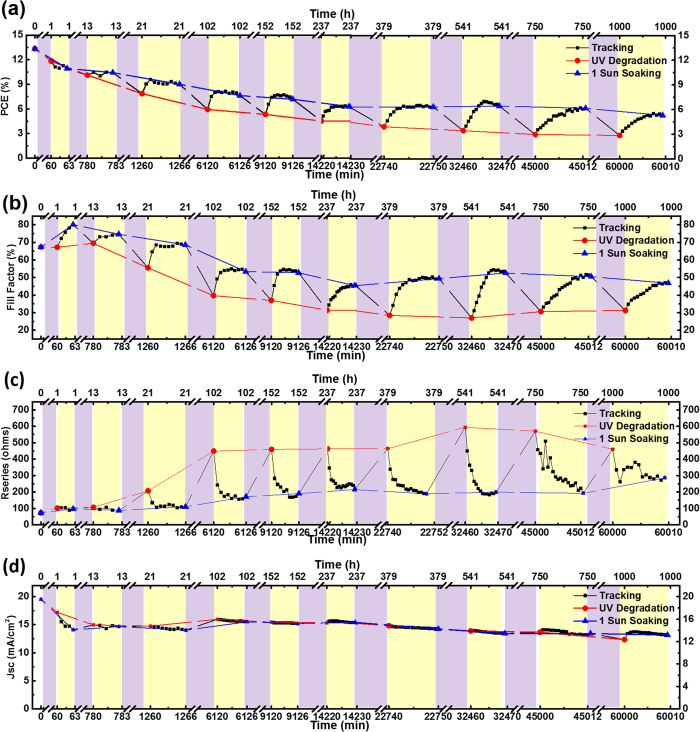
UV degradation/recovery cycle of (**a**) PCE, (**b**) FF, (**c**) R_s_, and (**d**) J_sc_ for device subjected to a range of UV exposure and 1-sun light illumination. Purple regions represent UV exposure and yellow regions represent 1-sun light illumination periods. The breaks used in this figure. Red (circle) symbol denotes values after UV exposure and blue (triangle) symbol denotes values after 1-sun light illumination. Black (square) symbol denotes values during 1-sun light illumination. Lines between the symbols are guide to the eyes. V_oc_ data is shown in Supplementary Fig. S5. Data for other devices showing similar tendency are presented in Supplementary Figs S6–10.

As previously mentioned in Fig. 1, UV degradation/recovery was repeated throughout the investigation. Figure 5 and Supplementary Fig. S5 present UV degradation/recovery during 1,000 h and at specific time. Degradation/recovery phenomenon of perovskite solar cells was also observed for other devices (Supplementary Figs S6–10). Supplementary Fig. S11a shows I-V curves for the pristine, UV-degraded, and partly recovered devices. Parameters are summarized in Supplementary Table S1. With 1-sun light soaking, PCE, FF, V_oc_, R_s_ and R_shunt_ (Figs S11b,c,d,f) underwent continuous recovery up to saturation. The J_sc_ (Supplementary Fig. S11d) exhibited a rapid increase in the first stage of 1-sun light soaking, followed by a continuous decrease.

## Discussion

Repeated UV degradation and 1-sun light soaking induced recovery attribute to the interface and bulk trap neutralization by photo-generated carriers. As a results of trap neutralization, electron extraction will be enhanced and this will improve V_oc_, R_s_, R_shunt_ and overall FF^57^,^58^,^59^,^60^,^61^ during 1-sun light soaking. Consequently, UV degradation/recovery cycle can be explained by repeated process of interface defects generation by UV light and neutralization by photo-generated carriers under 1-sun light soaking. During this cycle, the photocatalytic effect of the mesoporous TiO_2_ layer causes decomposition of the CH_3_NH_3_PbI_3_ perovskite to PbI_2_; degradation tendency can be seen in Fig. 5 (red line, UV degradation) and Fig. 2 40,44,45. As shown is Supplementary Fig. S11d, rapid recovery and continuous degradation of the J_sc_ during 1-sun light soaking can be explained by light-induced meta-stable trap formation at the perovskite bulk during 1-sun illumination. According to the literature, continuous 1-sun illumination will generate a quasi-static charge state by lattice distortion and phase separation. These states will be accumulated and coexist with photo-generated carriers, leading to current degradation during further 1-sun light soaking^22^,^62^,^63^,^64^.

When devices were exposed to UV light hundreds of hours, UV degradation/recovery mechanism seems to be changed. 650 h UV exposed devices were recovered with 1-sun light soaking and stored in the dark to more detail investigation. If recovery process is related to interface defects only, the system should return to its initial position because of the de-trapping of carriers as indicated in Fig. 1. Supplementary Fig. S12, shows very little change with storing in the dark (Ar atmosphere). This suggest longer UV exposure can change UV degradation/recovery mechanism. Light-induced meta-stable trap states^22^,^62^,^63^ and holes accumulation^65^ can have rules in UV degradation/recovery.

**Figure 6:**
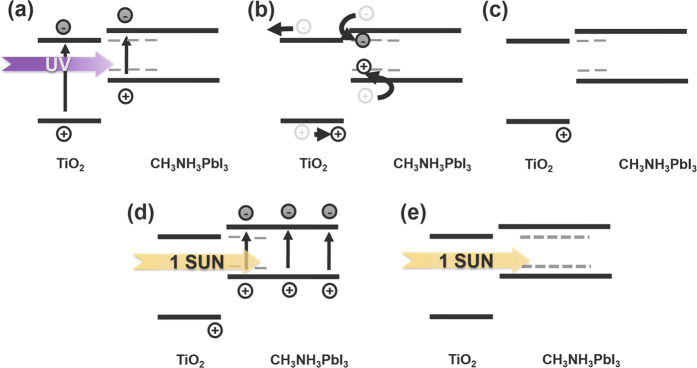
Schematic representation of the proposed mechanisms for UV degradation and recovery of perovskite solar cells.

Meta-stable trap states and charges are expected to accumulate primarily on the TiO_2_/perovskite interface. Supplementary Fig. S13a shows the UV-Vis transmittance profile of the perovskite device components in the UV region, in addition to the calculated relative UV light intensity inside of the perovskite device by the Lambert law of absorption. Based on the transmittance and absorption coefficients of CH_3_NH_3_PbI_3_^10^,^11^,^12^,^13^,^14^, Supplementary Fig. S13b gives the calculated UV light intensity decay. Based on the calculations shown in Supplementary Fig. S13b, we propose a possible mechanism for the UV degradation/recovery of the perovskite solar cells. As indicated in Fig. 6, because UV light cannot penetrate deep into the perovskite device, UV light will be absorbed close to the interface of perovskite/TiO_2_. As a results, meta stable trap-states and charges accumulation occur close to the mesoporous TiO_2_/perovskite interface. In this case, perovskite bulk has not enough electrons to change position with the holes allowing it to flow to the hole transport material (HTM) side. Such electron deficiency can result in trapped holes. The accumulated meta-stable trap states and charges will be generated in following UV irradiation. These traps and charges can extract electrons from the iodine anion (I^−^) in the CH_3_NH_3_PbI_3_ crystals. This will compromise the perovskite crystal structure, producing PbI_2_. Furthermore, residual traps and charges can also capture free electrons generated upon subsequent 1-sun light irradiation and can block the current flow, resulting in a transiently lowered solar cell performance with a low FF. 1-sun light irradiation will generate free carriers in whole regions of the perovskite light absorber, and these carriers can neutralize accumulated trap states and charges, resulting in the recovered device performance.

In this paper, degradation of mesoscopic CH_3_NH_3_PbI_3_ perovskite by UV light alone was evaluated. UV degradation occurred in the absence of moisture and other wavelengths of light, likely due to the photocatalytic effect of mesoporous TiO_2_, accumulated interface trap states and charges. A current bounce-back phenomenon was attributed to the trap passivation and enhanced electron extraction by UV degradation by-product PbI_2_. The UV-degraded cell performance was subsequently recovered by 1-sun light soaking, with >60% of the initial degradation being recovered, and this degradation/recovery cycle could be repeated. The mainly degraded/recovered parameter was the fill factor. UV degradation/recovery phenomenon was considered as the neutralization and resolving of accumulated traps states and charges by free carriers generated by 1-sun light soaking.

## Methods

FTO glass substrates (7 Ω/sq) were rinsed with acetone, ethanol, and isopropyl alcohol (IPA) with sonication. The substrate was then subjected to UV ozone treatment for 30 min. A compact TiO_2_ layer was spin-coated with a 0.15 M titanium diisopropoxide bis(acetylacetonate) solution (75 wt%, Sigma-Aldrich) in 1-butanol (ACS reagent, ≥99.4%, Sigma-Aldrich), and the resulting TiO_2_ layer was heat-treated for 15 min at 500 °C. Subsequently, a mesoporous TiO_2_ solution was prepared by mixing TiO_2_ paste (18NR-T Transparent titania paste, Dyesol), terpineol (mixture of isomers, anhydrous, Sigma-Aldrich), and ethyl alcohol (pure, 200 proof, anhydrous, Sigma-Aldrich) in a 1:4:2 wt% ratio, followed by heat treatment at 550 °C for 60 min. The CH_3_NH_3_PbI_3_ perovskite layer was then prepared according to a previously reported sequential deposition method^17^. Lead(II) iodide powder (PbI_2_, 99.9985% metal basis, Alfa Aesar) was dispersed in *N*,*N*-dimethylformamide (DMF, anhydrous, 99.8%, Sigma Aldrich) in a 1.1:1 molar ratio. After spin-coating PbI_2_ on the mesoporous TiO_2_ film, annealing was carried out first at 40 °C for 3 min and then at 75 °C for 10 min. A dipping solution was prepared; the solution contained methylammonium iodide (MAI, 0.9 g, Dyesol) dispersed in isopropyl alcohol (IPA, 10 mL, anhydrous, 99.5%, Sigma Aldrich). The PbI_2_ film was immersed in the solution prior to annealing at 100 °C for 30 min. 2,2′,7,7′-Tetrakis(*N*,*N*-di-*p*-methoxyphenyl-amine)-9,9′-spirobifluorene (Spiro-MeOTAD, HANALINTECH), doped with bis(trifluoromethane)sulfonimide lithium salt (Li-TSFI, 99.95% trace metals basis, Sigma-Aldrich) was used as an HTM. A solution of the HTM was prepared by dissolving Spiro-MeOTAD (72.3 mg) in chlorobenzene (1 mL, 99.8% Sigma-Aldrich) containing 4-tert-butyl pyridine (28.8 μL, 96%, Sigma-Aldrich) and Li-TSFI solution (17.5 μL, 520 mg Li-TSFI in 1 mL anhydrous acetonitrile (99.8%, Sigma-Aldrich)). Finally, a 100 nm Au electrode was deposited by thermal evaporation.

The UV degradation experiment was conducted in a glove box at 25 °C under an inert (Ar) atmosphere at <0.5 ppm humidity. Regeneration of the glove box atmosphere was performed twice to control the humidity and to remove traces of solvent. The perovskite solar cells were exposed to 4 W, 365 nm (VL-4.LC, VILBER LOURMAT, 350 μW·cm^−2^ at 15 cm) UV light for 1,000 h, 2 cm above the samples. UV light irradiation at 2 cm apart from UV lamp shows 19 Lux ( = 7.6 mW·cm^−2^ in case of Sunlight), which was measured by Testo 540-Lux Meter.

1-Sun light illumination for the recovery experiment was performed under 100 mW·cm^−2^ irradiation at room temperature and 25% average relative humidity. The device temperature was approximately 40 °C after 10 min 1-sun light irradiation. All devices were illuminated with a 0.075 cm^2^ mask.

### Device characterization

The UV-induced degradation of the perovskite solar cells was studied over a range of time intervals. The cells were removed from the glove box at specifically defined times and characterized under air at room temperature and 25% average relative humidity.

The light absorbance and transmittance were measured by UV-Vis spectroscopy (JASCO V-670 UV/Vis NIR spectrophotometer), and X-ray diffractometry (XRD, SmartLab, Rigaku) was carried out using CuKα radiation (1.54 nm). The solar cell parameters of the devices were measured using a solar simulator (WXS-155S-10, AG1.5G, WACOM) under 100 mW·cm^−2^ irradiation. All devices had an active area of 0.125 cm^2^ and all light I-V measurements were performed using a 0.075 cm^2^ mask. The scan direction was open circuit voltage to short circuit current (i.e., reverse direction) and the voltage setting time was 200 ms. External quantum efficiency measurements were conducted using a 100 Hz chopping frequency.

Electrochemical Impedance spectroscopy measurements were performed with IVIUM, IviumStat by applying the alternative signal of 10 mV at ten points per decade in the frequency range of 10^6^~1 Hz with DC voltage set point under dark conditions. The obtained Nyquist plots were modelled by using Z-View software.

The μ-PLS and μ-LBIC mappings were performed with a photoluminescence spectroscopy setup using a confocal microscope, illuminating the samples from the glass side^66^,^67^. The point-shaped excitation and detection allows for diffraction limited resolution when using an objective lens with a low numerical aperture (<0.1). In this case, an objective lens with a numerical aperture (NA) of 0.26 was used in order to enhance the detection spot size and therefore allow for a reduction of the integration time. For both measurements, an excitation wavelength of 640 nm and an illumination intensity of 3 μW, with a laser spot size of 430 μm^2^, were chosen. For detection of the PL signal, a silicon line CCD combined with a grating spectrometer was used, providing the PL spectrum for each measurement spot. In order to suppress the excitation light, a 700 nm longpass filter was applied. Both the spectral position and the peak height were obtained from a Gaussian fit of the measurement data. As the local light beam-induced current is very low, the signal was amplified using a low noise preamplifier.

## Additional Information

**How to cite this article**: Lee, S.-W. *et al*. UV Degradation and Recovery of Perovskite Solar Cells. *Sci. Rep.* **6**, 38150; doi: 10.1038/srep38150 (2016).

**Publisher's note:** Springer Nature remains neutral with regard to jurisdictional claims in published maps and institutional affiliations.

## Supplementary Material

Supplementary Information

## Figures and Tables

**Table 1:** Solar cell parameters during UV exposure (all values are an average of data obtained from six cells).

UV Exposure Time [hour]	V_oc_ [V]	J_sc_ [mA∙cm^−2^]	FF [%]	PCE [%]
Pristine	1.038 (1.03 Stored in Dark)	19.71 (19.69 Stored in Dark)	69.02 (68.78 Stored in Dark)	14.12 (13.97 Stored in Dark)
1	0.992	15.26	77.21	11.64
13	0.986	15.87	70.39	11.03
21	0.991	16.32	68.08	11.07
102	0.988	17.35	53.00	9.19
152	0.980	17.60	51.16	8.94
237	0.990	17.47	52.67	9.32
379	0.971	17.16	48.14	8.28
541	0.942	15.83	38.96	5.89
750	0.853	15.53	39.90	5.41
1000	0.846 (1.053 Stored in Dark)	14.58 (20.08 Stored in Dark)	41.94 (65.47 Stored in Dark)	5.35 (13.84 Stored in Dark)
