# Supplementary material for: UV Degradation and Recovery of Perovskite Solar Cells
Source: Sci Rep. 2016 Dec 2;6:38150. doi: 10.1038/srep38150 (PMC5133559; doi:10.1038/srep38150)
Supplement: Supplementary Information [file srep38150-s1.pdf]

Supplementary Information

UV Degradation and Recovery of Perovskite Solar Cells

Sang-Won Lee[1], Seongtak Kim[1], Soohyun Bae[1], Kyungjin Cho[1], Taewon Chung[1], Laura E. Mundt[2], Seunghun Lee[1,2], Sungeun Park[1,2], Hyomin Park[1,2], Martin C. Schubert[2], Stefan W. Glunz[2,3], Yohan Ko[4], Yongseok Jun[4], Yoonmook Kang[5], Hae-Seok Lee[5], and Donghwan Kim[1,*]

[1] Korea University, Department of Materials Science and Engineering, Seoul, 136-713, Republic of Korea
[2] Fraunhofer Institute for Solar Energy Systems ISE, Freiburg, 79110, Germany
[3] University Freiburg, Laboratory for Photovoltaic Energy Conversion, Freiburg, 79110, Germany
[4] Konkuk University, Department of Materials Chemistry and Engineering, Seoul, 143-701, Republic of Korea
[5] KU·KIST Green School, Graduate School of Energy and Environment, Seoul, 136-713, Republic of Korea

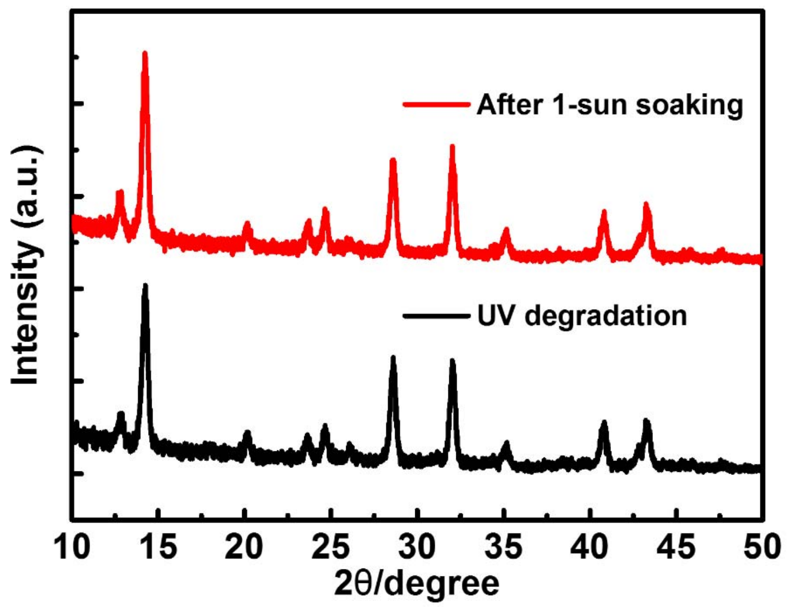

Supplementary Fig. S1 X-ray diffraction patterns of UV degraded sample before and after 1-sun light soaking.

.

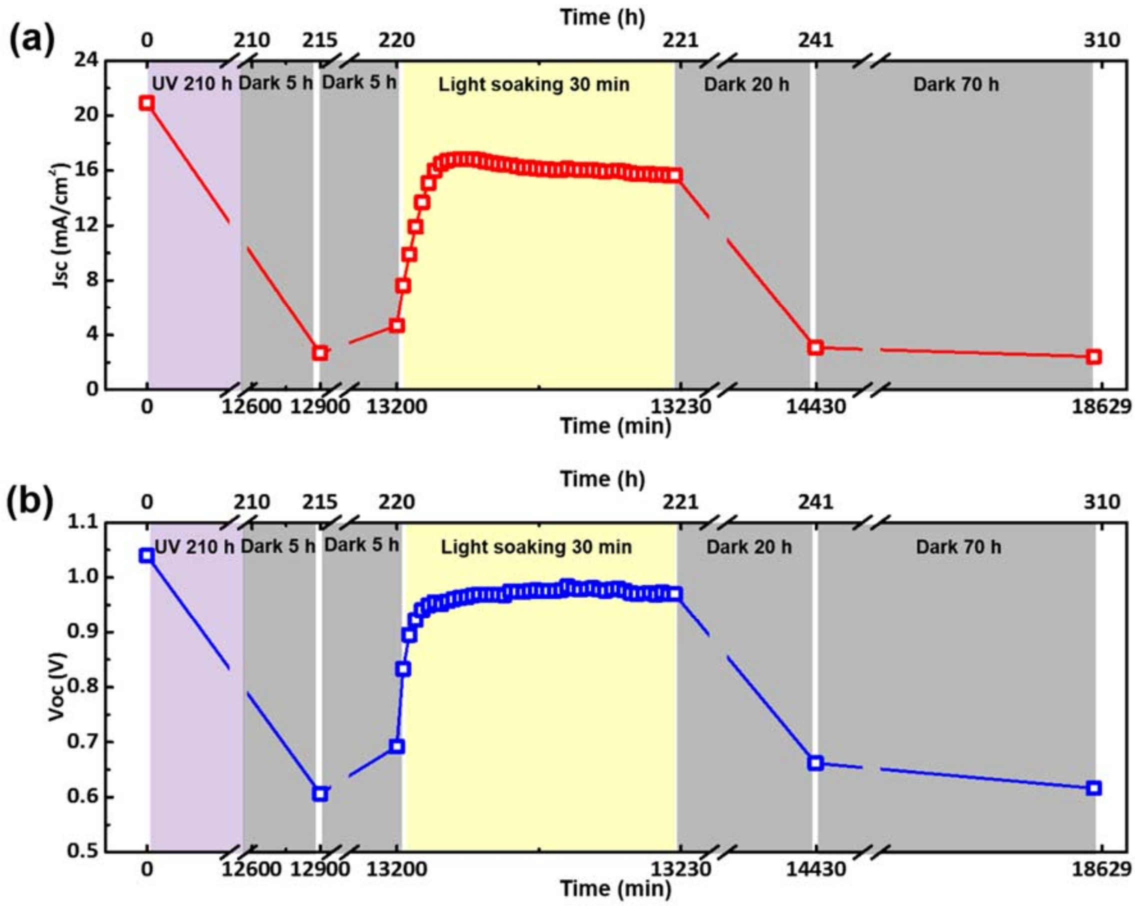

Supplementary Fig. S2 (a) J_{sc} and (b) V_{oc} of a device with denoted light conditions. Device firstly exposed to UV light 210 h and then rested in dark 5 h and measured. After measurements, device rested in dark 5 h again and measured with 1-sun light soaking. Measurements were conducted with 40 s intervals during light soaking. Device was measured again after 20 h, and 70 h rested in dark. UV light exposure and resting in dark were conducted under inert gas atmosphere. 1-sun light soaking was conducted under air condition.

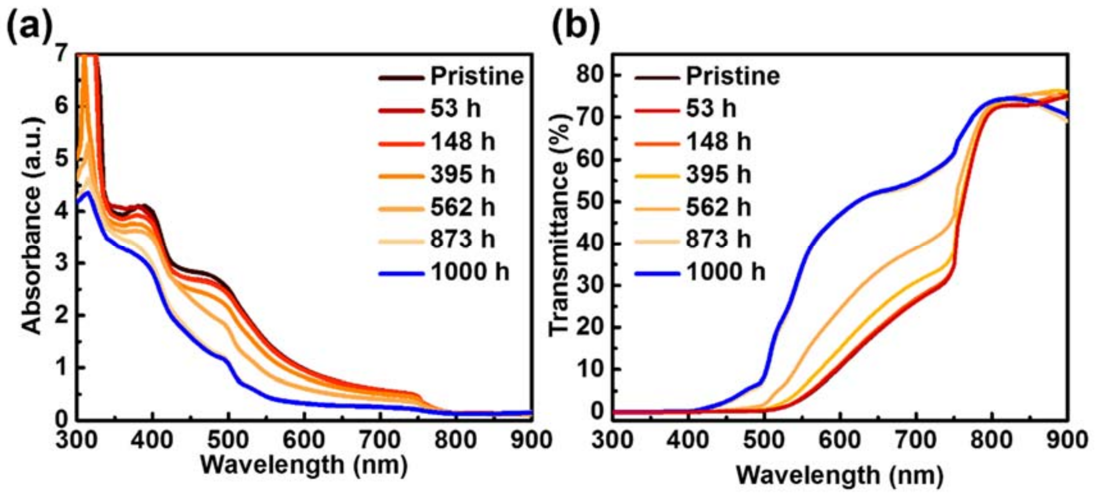

Supplementary Fig. S3 UV-Visible (a) light absorbance (b) transmittance of pristine and UV degraded devices measured at 300–900 nm. Times shown denote UV exposure times.

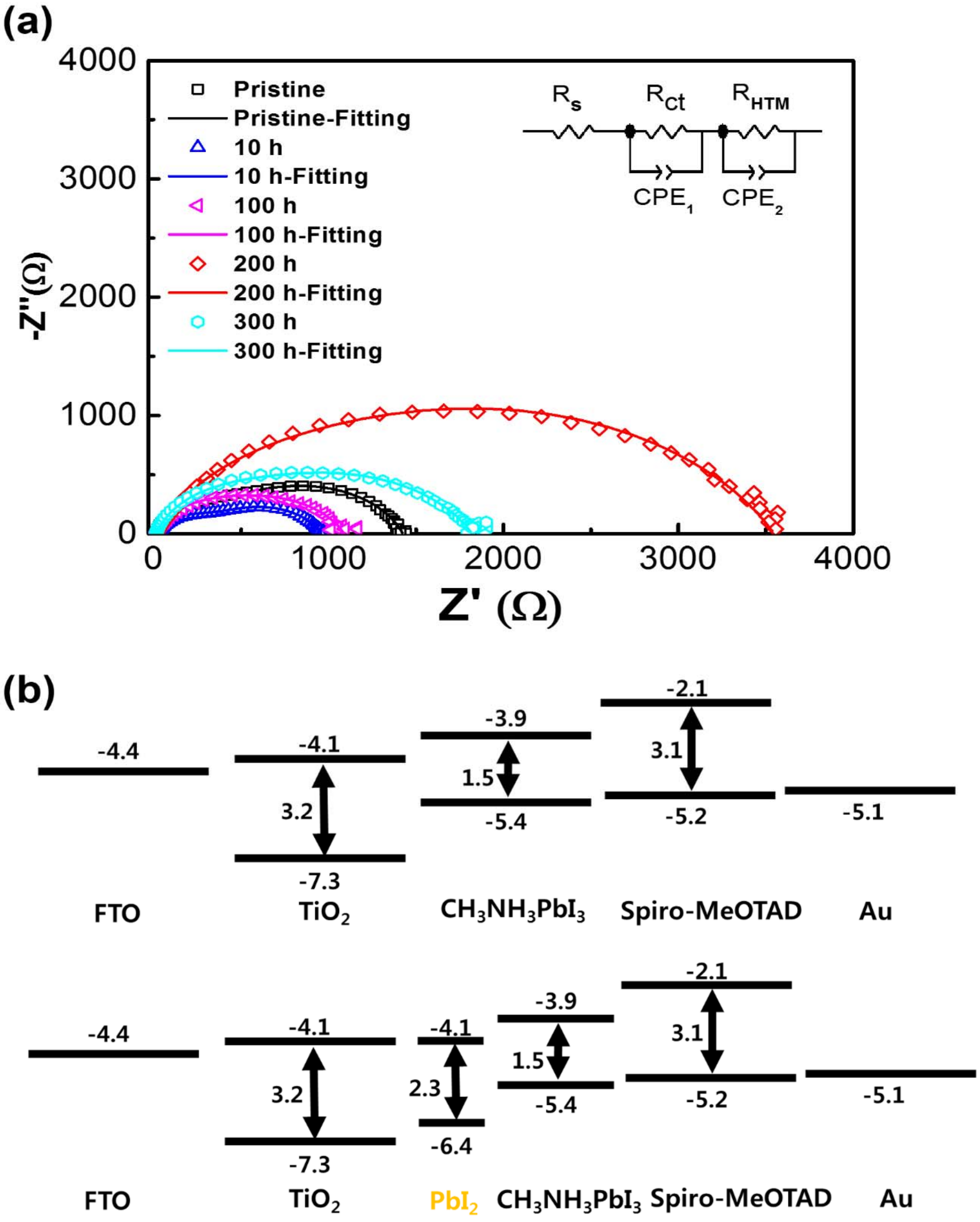

Supplementary Fig. S4 (a) Nyquist plots of UV exposed perovskite solar cells for different times and (b) possible band structure change with PbI_2 formation which can be beneficial for charge transport[1]. EIS measurements obtained under dark conditions at a 0.8 bias voltage. Inset represents the equivalent circuit for fitting EIS: R_s (series resistance), R_{ct} (recombination resistance) and R_{HTM} (HTM resistance) in parallel with each chemical capacitance CPE1, CPE2. The Nyquist plots of these perovskite solar cells shows two main arcs. The high frequency arc in the Nyquist plots is attributed to R_{HTM}. Recombination at TiO_2/Perovskite interfaces, R_{ct}, is displayed in lower frequency region. As depicted in Nyquist plots, R_{ct} is reduced after 10h UV exposure. However, the R_{ct} is recovered as increasing the UV exposure time. This enhancement of R_{ct} as following UV exposure suggests that the generation of PbI_2 at TiO_2/Perovskite interfaces suppress charge recombination, which may passivate defect sites exist at the interfaces generated during UV degradation[2, 3].

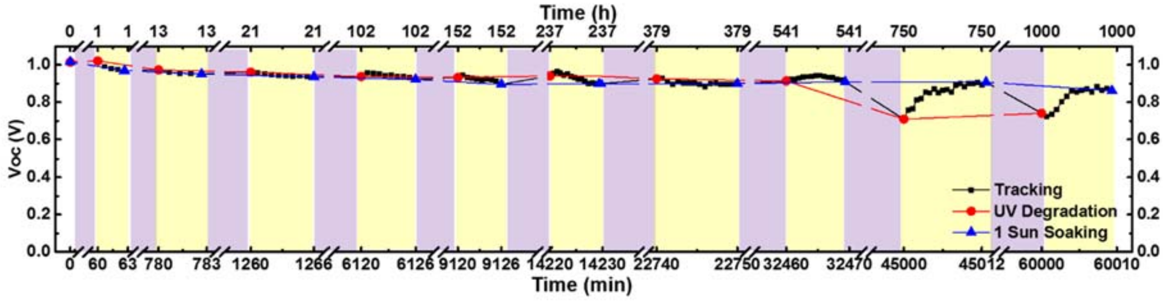

Supplementary Fig. S5 V_{oc} degradation/recovery cycle of device represented in Fig. 5 (of the main article) over a range of UV exposure and 1-sun light irradiation times. The breaks are used to indicate two different time scales. Red (circle) symbol denotes values after UV exposure and blue (triangle) symbol denotes values after 1-sun light irradiation. Black (square) symbol denotes values during 1-sun light irradiation. Lines between the symbols are a guide to the eyes. Purple region represents UV exposure and yellow region represents 1-sun light irradiation region.

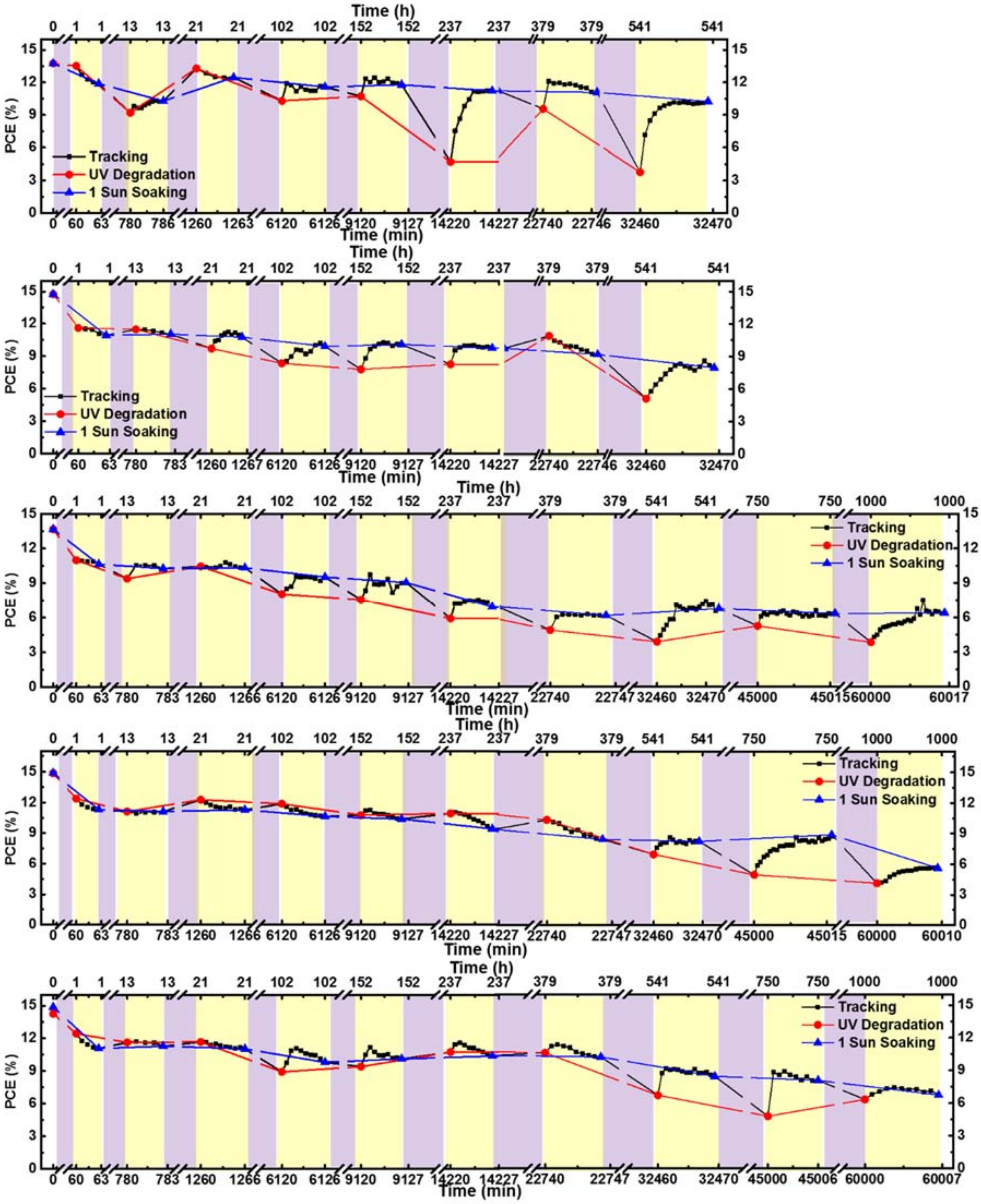

Supplementary Fig. S6 PCE degradation/recovery cycle of five devices over a range of UV exposure and 1-sun light irradiation times**.** The breaks are used in this figure to indicate two different time scales. Red (circle) symbol denotes values after UV exposure and blue (triangle) symbol denotes values after 1-sun light irradiation. Black (square) symbol denotes values during 1-sun light irradiation. Lines between the symbols are a guide to the eyes. Purple region represents UV exposure and yellow region represents 1-sun light irradiation region.

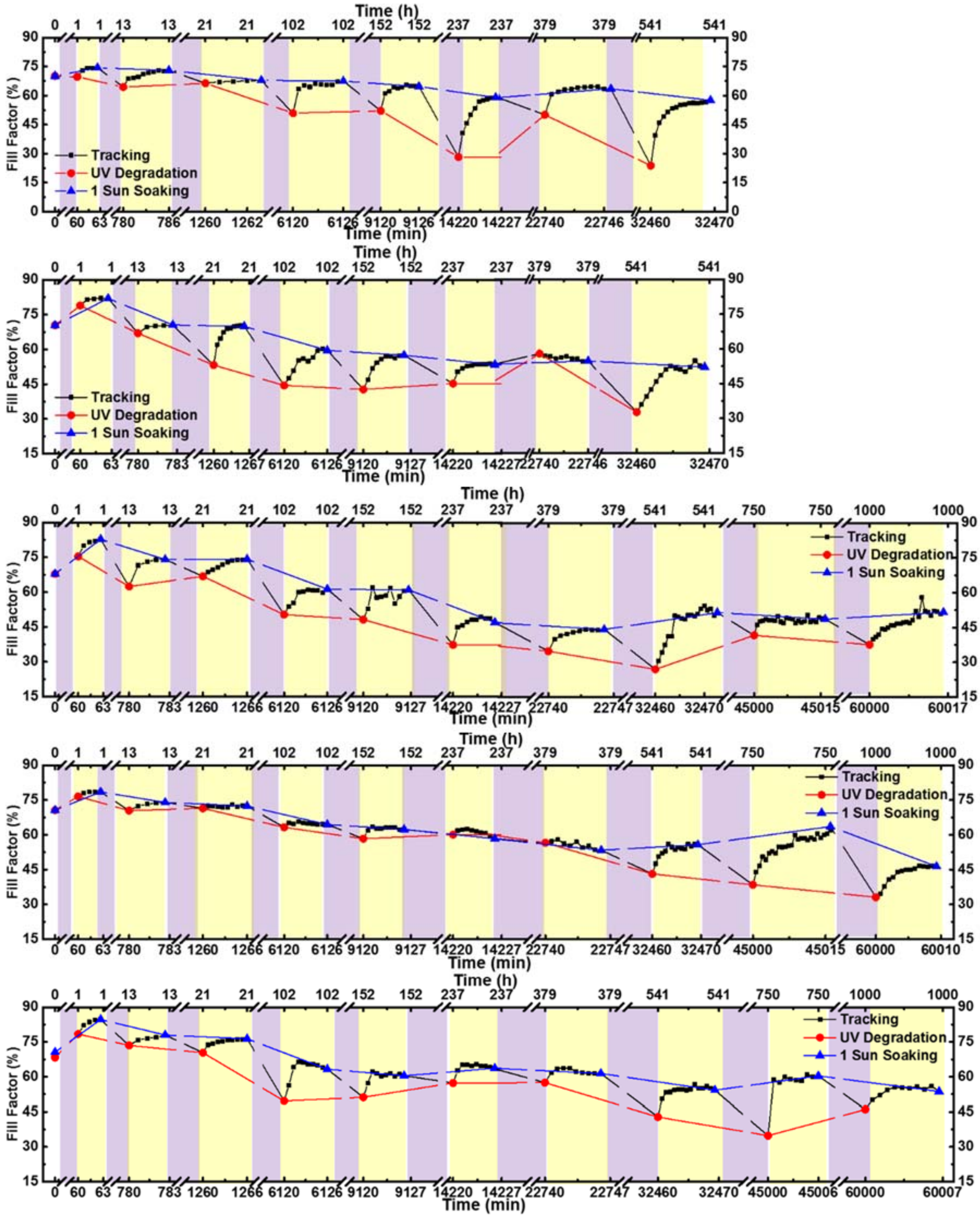

Supplementary Fig. S7 FF degradation/recovery cycle of five devices over a range of UV exposure and 1-sun light irradiation times. The breaks used in this figure indicate two different time scales. Red (circle) symbol denotes values after UV exposure and blue (triangle) symbol denotes values after 1-sun light irradiation. Black (square) symbol denotes values during 1-sun light irradiation. Lines between symbols are a guide to the eyes. Purple region represents UV exposure and yellow region represents 1-sun light irradiation region.

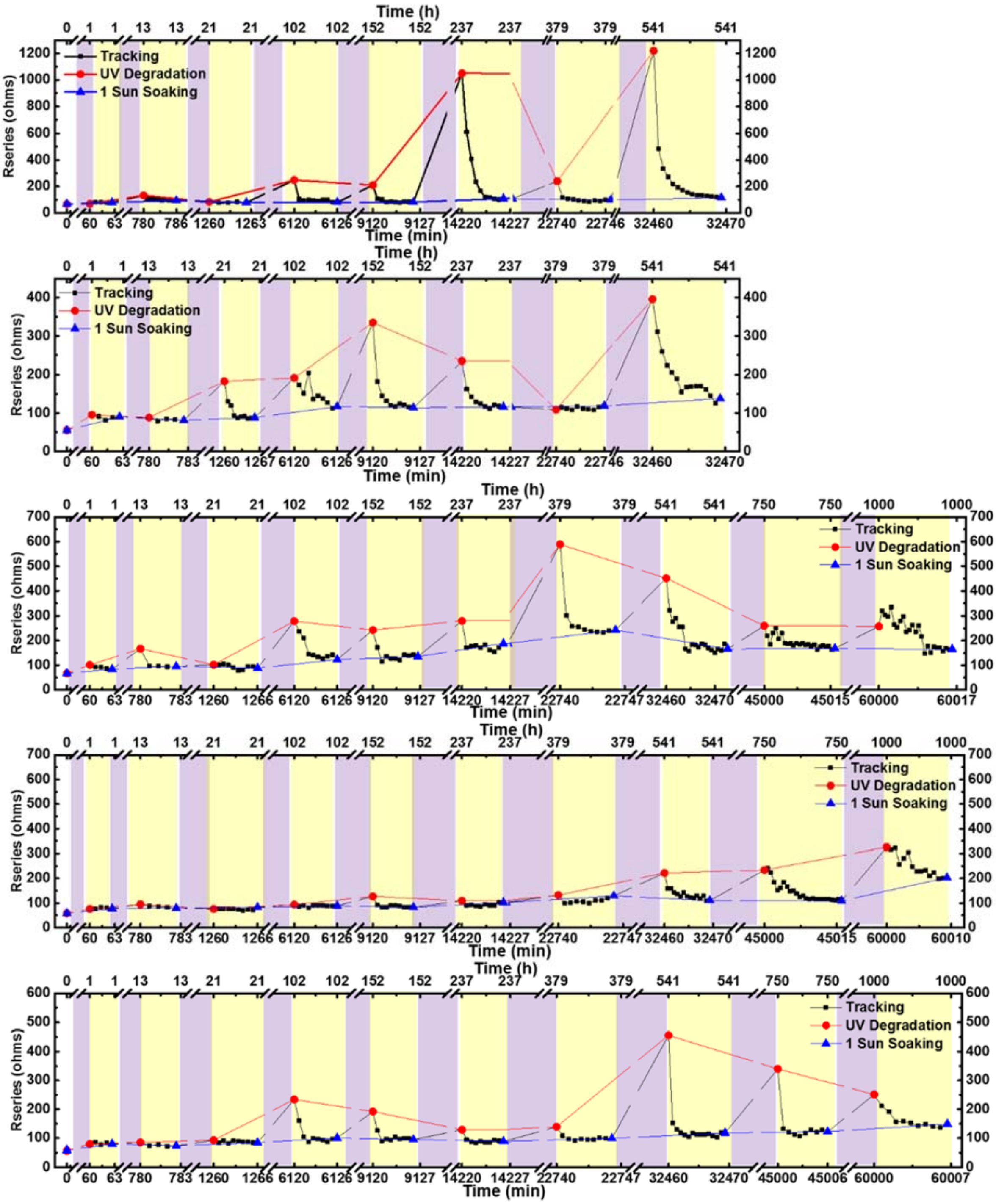

Supplementary Fig. S8 R_s degradation/recovery cycle of five devices over a range of UV exposure and 1-sun light irradiation times. The breaks used in this figure denote two different time scales. Red (circle) symbol denotes values after UV exposure and blue (triangle) symbol denotes values after 1-sun light irradiation. Black (square) symbol denotes values during 1-sun light irradiation. Lines between the symbols are a guide to the eyes. Purple region represents UV exposure and yellow region represents 1-sun light irradiation region.

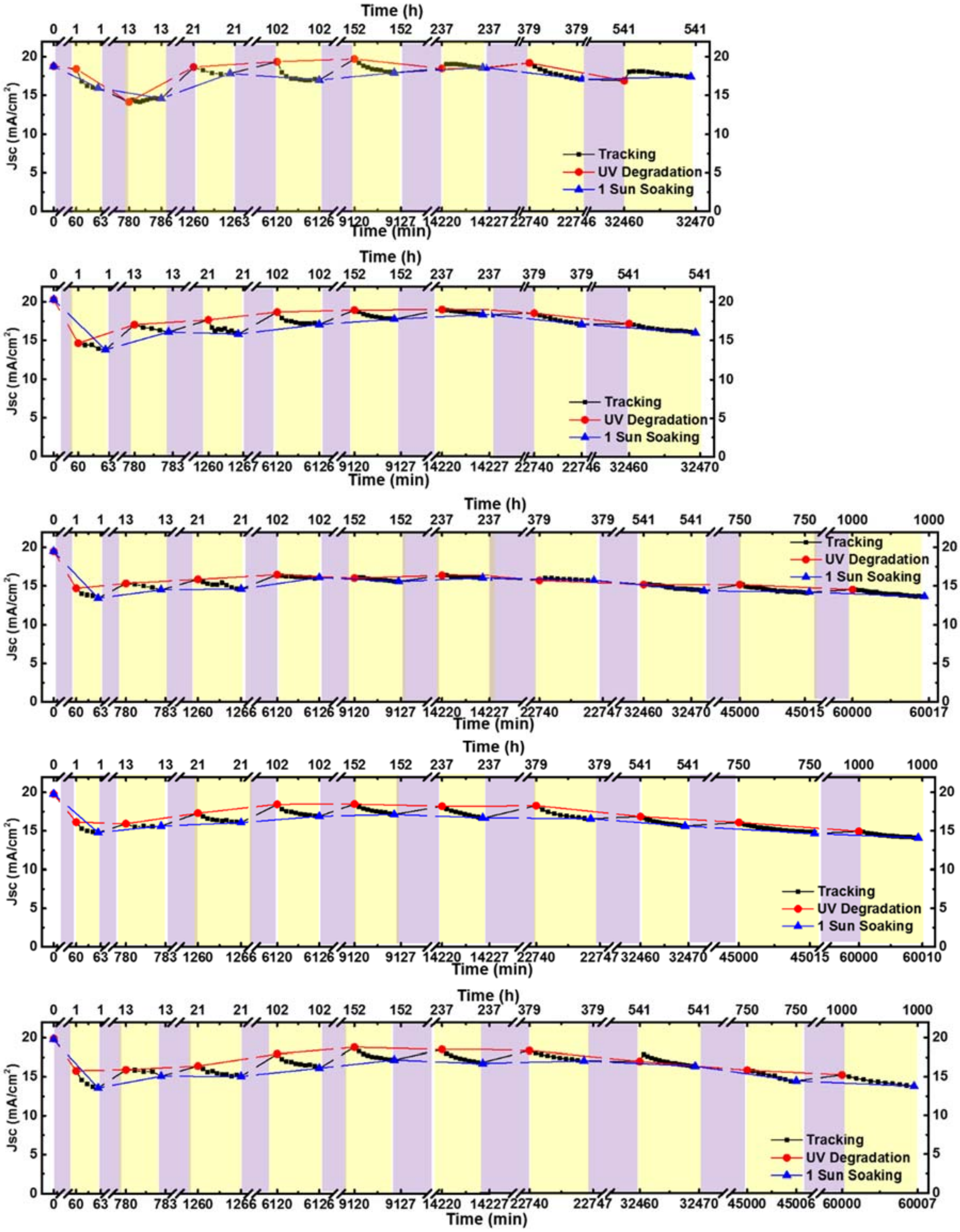

Supplementary Fig. S9 J_{sc} degradation/recovery cycle of five devices over a range of UV exposure and 1-sun light irradiation times. The breaks used in this figure denote two different time scales. Red (circle) symbol denotes values after UV exposure and blue (triangle) symbol denotes values after 1-sun light irradiation. Black (square) symbol denotes values during 1-sun light irradiation. Lines between the symbols are a guide to the eyes. Purple region represents UV exposure and yellow region represents 1-sun light irradiation region.

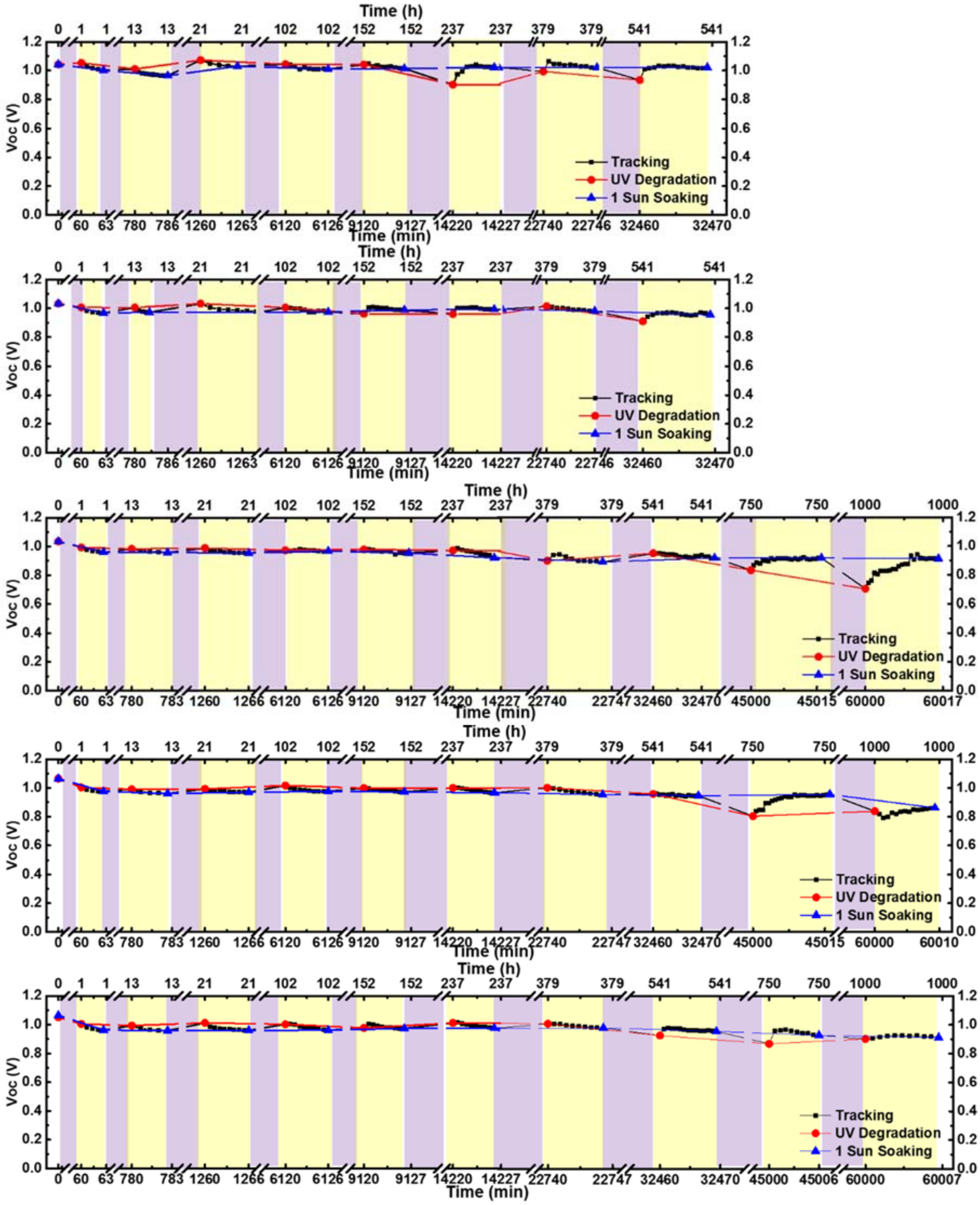

Supplementary Fig. S10 V_{oc} degradation/recovery cycle of five devices over a range of UV exposure and 1-sun light irradiation times. The breaks used in this figure denote two different time scales. Red (circle) symbol denotes values after UV exposure and blue (triangle) symbol denotes values after 1-sun light irradiation. Black (square) symbol denotes values during 1-sun light irradiation. Lines between the symbols are a guide to the eyes. Purple region represents UV exposure and yellow region represents 1-sun light irradiation region.

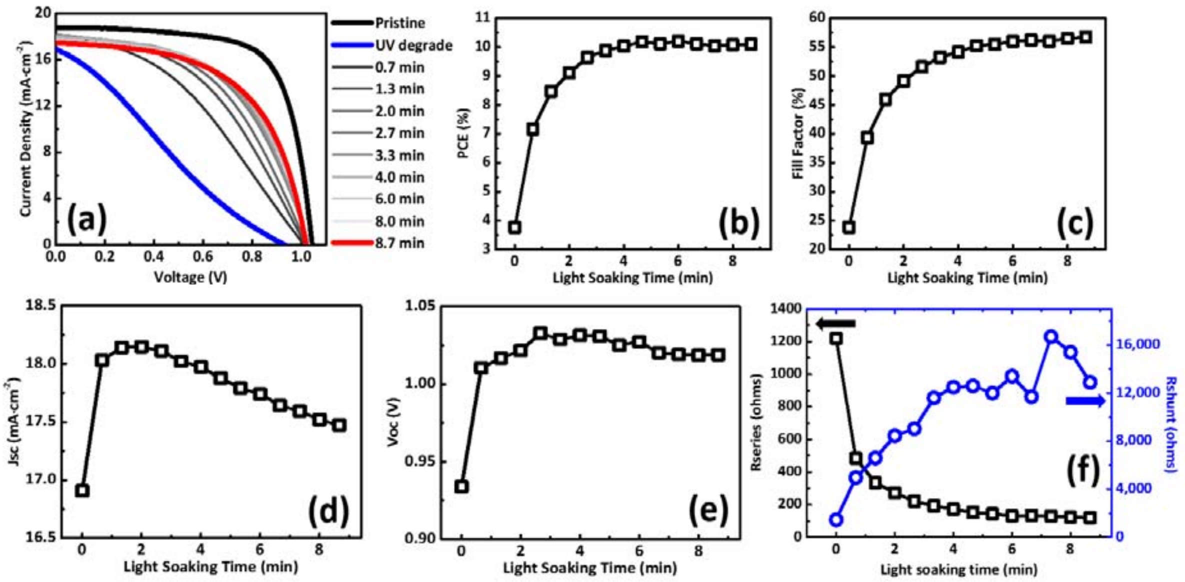

Supplementary Fig. S11 1-Sun light soaking induced recovery of the UV degraded perovskite solar cells. (a) Light I-V curve, (b) PCE, (c) FF, (d) J_{sc}, (e) V_{oc}, (f) R_s, and R_{shunt} with continuous light soaking at 1-sun intensity (AM 1.5G, 100 mW·cm^{-2}) for about 9 min. The voltage setting time was 200 m·s, the active area was 0.125 cm^2, and a mask of 0.075 cm^2 was used for the measurements. Measurements were carried out in the open circuit voltage to short circuit current direction (i.e., reverse direction). Measurements were performed at 40 s intervals. Series and shunt resistance were deduced from V_{oc} and J_{sc} slopes and calculated automatically by measurement software.

Supplementary Table. S1 Solar cell parameters during light soaking under 1-sun about 9 min (100 $mW \cdot cm^{-2}$ illumination)

Light soaking time [min]	V_{oc} [V]	J_{sc} [$mA \cdot cm^{-2}$]	FF [%]	Series Resistance [Ω]	Shunt Resistance [Ω]	PCE [%]
Pristine	1.043	18.77	70.15	67.1	-20800	13.74
UV degraded	0.934	16.91	23.87	1220	1460	3.77
0.7	1.011	18.03	39.33	484	4970	7.17
1.3	1.017	18.14	45.95	334	6590	8.47
2.0	1.022	18.15	49.14	272	8450	9.11
2.7	1.033	18.11	51.6	220	9040	9.65
3.3	1.029	18.02	53.27	192	11600	9.88
4.0	1.032	17.97	54.12	171	12500	10.03
4.7	1.031	17.88	55.24	153	12600	10.18
5.3	1.025	17.79	55.46	143	12000	10.11
6.0	1.027	17.74	55.97	132	13400	10.20
6.7	1.020	17.64	56.2	134	11700	10.11
7.3	1.019	17.59	55.98	130	16700	10.04
8.0	1.019	17.52	56.48	124	15400	10.08
8.7	1.019	17.47	56.71	120	12900	10.10

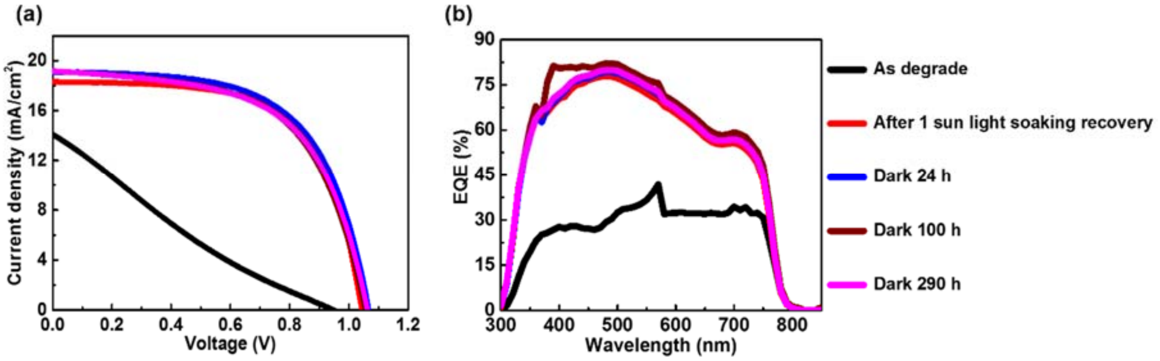

Supplementary Fig. S12 Light I-V and EQE data. Devices were firstly degraded with UV light and recovered with 1-sun light soaking. After recovery, devices were stored in a glove box without any illumination. After the times denoted above, devices were removed from glove box and measured again.

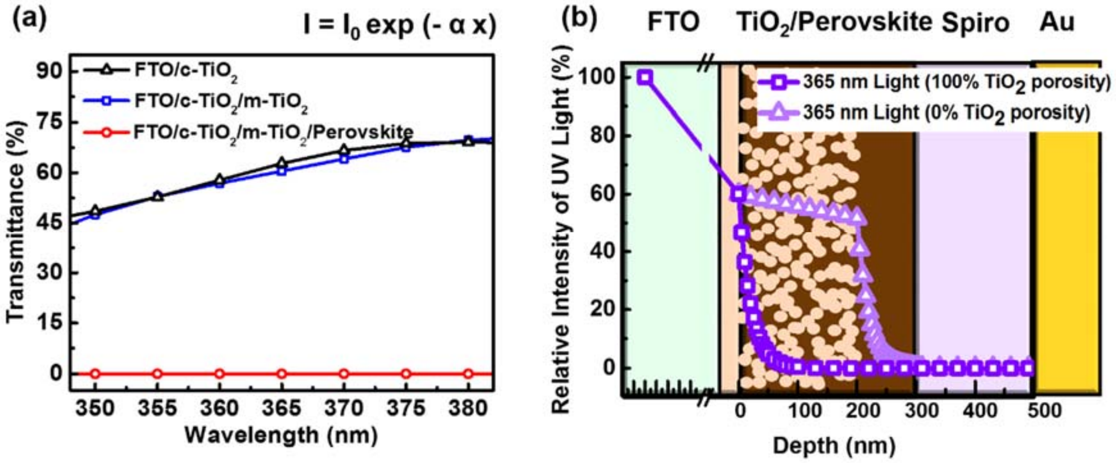

Supplementary Fig. S13 (a) Light transmittance of the perovskite solar cell components and (b) relative intensity of UV light calculated based on the intensity decay equation. The relative intensity inside the perovskite was plotted by using device figures with a real scale ratio. Lambert law of absorption: $I = I_0 \exp(-ax)$, where I: intensity of light, I_0: incident light intensity, a: absorption coefficient, and x: penetration depth of light. A light absorption coefficient of 5.0×10^5 cm^{-1} was used for the $CH_3NH_3PbI_3$ perovskite and 0, 100% porosity of mesoporous TiO_2 was assumed and calculated[4-11].

References

1. Cao, Duyen H., et al. "Remnant PbI2, an unforeseen necessity in high-efficiency hybrid perovskite-based solar cells? a)." *Apl Materials* 2.9 091101, doi: 10.1063/1.4895038 (2014).
2. Gonzalez-Pedro, Victoria, et al. General working principles of $CH_3NH_3PbX_3$ perovskite solar cells. *Nano letters* 14.2 888-893, doi: 10.1021/nl404252e (2014).
3. Mora-Seró, Iván, et al. Impedance spectroscopy characterisation of highly efficient silicon solar cells under different light illumination intensities. *Energy & Environmental Science* 2.6 678-686, doi: 10.1039/B812468J (2009).
4. Kim, H. S. *et al.* Lead iodide perovskite sensitized all-solid-state submicron thin film mesoscopic solar cell with efficiency exceeding 9%. *Sci. Rep.* **2,** doi: 10.1038/Srep00591 (2012).
5. Chen, C. W. *et al.* Optical properties of organometal halide perovskite thin films and general device structure design rules for perovskite single and tandem solar cells. *J. Mater. Chem. A* **3,** 9152–9159, doi :10.1039/c4ta05237d (2015).
6. Lin, Q. Q., Armin, A., Nagiri, R. C. R., Burn, P. L. & Meredith, P. Electro-optics of perovskite solar cells. *Nat. Photonics* **9,** 106–112, doi: 10.1038/Nphoton.2014.284 (2015).
7. Loper, P. *et al.* Complex refractive index spectra of $CH_3NH_3PbI_3$ perovskite thin films determined by spectroscopic ellipsometry and spectrophotometry. *J. Phys. Chem. Lett.* **6,** 66–71, doi: 10.1021/jz502471h (2015).
8. Xie, Z. *et al.* Refractive index and extinction coefficient of $CH_3NH_3PbI_3$ studied by spectroscopic ellipsometry. *Opt. Mater. Express* **5,** 29–43, doi: 10.1364/Ome.5.000029 (2015).
9. Ni, M., Leung, M. K. H., Leung, D. Y. C. & Sumathy, K. An analytical study of the porosity effect on dye-sensitized solar cell performance. *Sol. Energ. Mat. Sol. C* **90,** 1331–1344, doi: 10.1016/j.solmat.2005.08.006 (2006).
10. Lee, J. J., Coia, G. M. & Lewis, N. S. Current density versus potential characteristics of dye-sensitized nanostructured semiconductor photoelectrodes. 1. Analytical expressions. *J. Phys. Chem. B* **108,** 5269–5281, doi: 10.1021/jp035194u (2004).
11. Lee, J. J., Coia, G. M. & Lewis, N. S. Current density versus potential characteristics of dye-sensitized nanostructured semiconductor photoelectrodes. 2. Simulations. *J. Phys. Chem. B* **108,** 5282–5293, doi: 10.1021/jp035195m (2004).
